# Author Correction: Passive eDNA collection enhances aquatic biodiversity analysis

**DOI:** 10.1038/s42003-021-01994-6

**Published:** 2021-04-06

**Authors:** Cindy Bessey, Simon Neil Jarman, Tiffany Simpson, Haylea Miller, Todd Stewart, John Kenneth Keesing, Oliver Berry

**Affiliations:** 1 Commonwealth Scientific and Industrial Research Organisation, Indian Oceans Marine Research Centre, Oceans and Atmosphere, Crawley, WA Australia; 2 Commonwealth Scientific and Industrial Research Organization, Indian Oceans Marine Research Centre, Environomics Future Science Platform, Crawley, WA Australia; 3 UWA Oceans Institute, University of Western Australia, Crawley, WA Australia; 4 School of Biological Sciences and the UWA Oceans Institute, University of Western Australia, Crawley, WA Australia; 5 eDNA Frontiers, Trace and Environmental DNA Laboratory, School of Molecular and Life Sciences, Curtin University, Perth, WA Australia; 6 Bass Marine Pty Ltd, Port Denison, WA Australia

**Keywords:** PCR-based techniques, Biodiversity

Correction to: *Communications Biology* 10.1038/s42003-021-01760-8, published online 22 February 2021.

The original version of this Article contained an error in the spelling of the authors Cindy Bessey, Simon Neil Jarman, Tiffany Simpson, Haylea Miller, Todd Stewart, John Kenneth Keesing and Oliver Berry, which were incorrectly given as Bessey Cindy, Jarman Simon Neil, Simpson Tiffany, Miller Haylea, Stewart Todd, Keesing John Kenneth and Berry Oliver.

This has now been corrected in both the PDF and HTML versions of the Article.

