# Supplementary material for: Passive eDNA collection enhances aquatic biodiversity analysis
Source: Commun Biol. 2021 Feb 22;4:236. doi: 10.1038/s42003-021-01760-8 (PMC7900116; doi:10.1038/s42003-021-01760-8)
Supplement: Supplementary file 2 — Supplemental Information [file 42003_2021_1760_MOESM2_ESM.pdf]

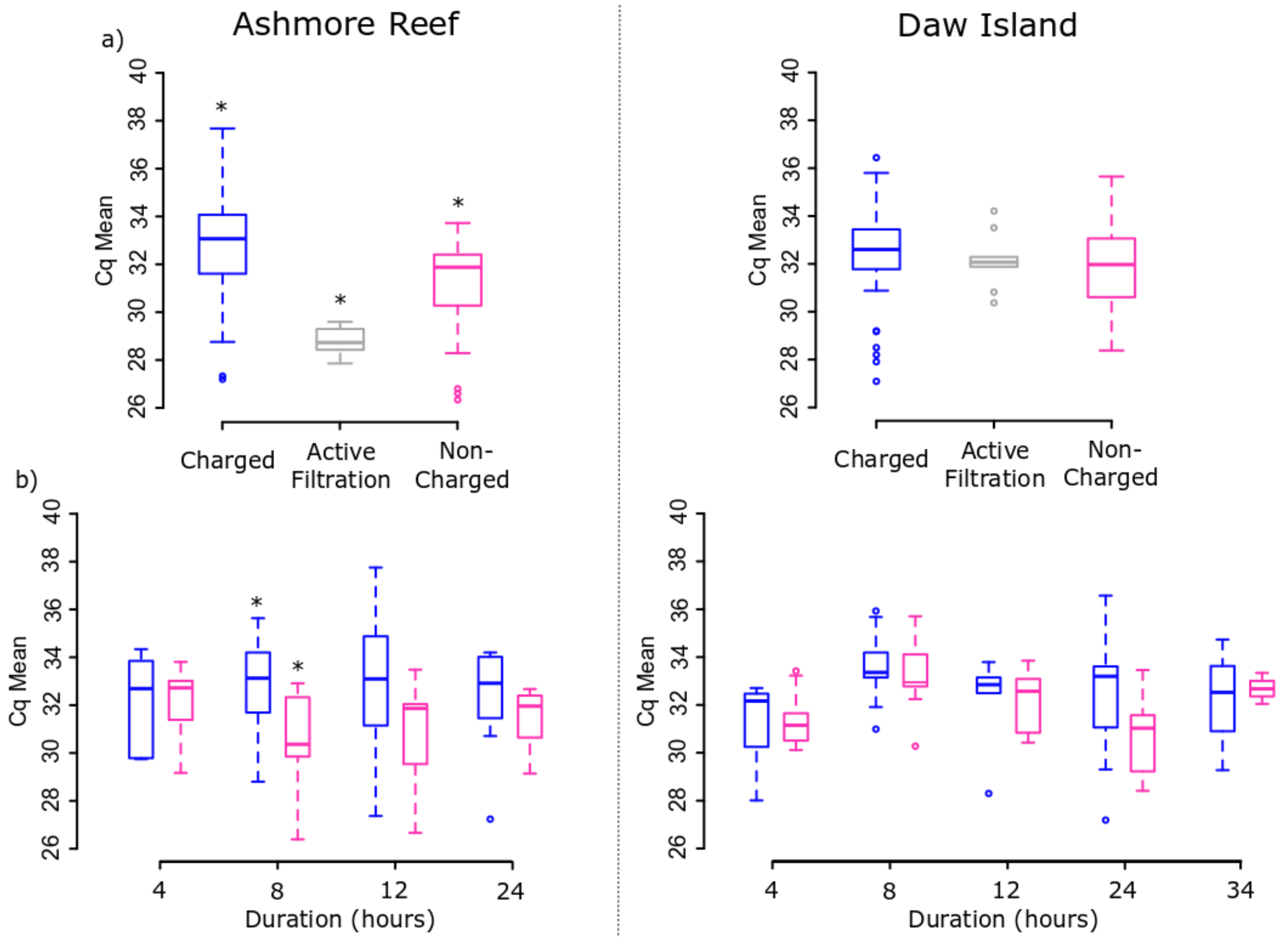

Figure S1. Boxplots of Cq mean values used as indication of initial DNA copy number at Ashmore Reef (charged n=33, active filtration n=9, and non-charged n=39), and , and Daw Island (charged n=38, active filtration n=9, and non-charged n=31), respectively, by treatment and b) by submersion duration and treatment (Ashmore Reef, charged: 4 hours n=6, 8 hours n=9, 12 hours n=8, 24 hours n=8, noncharged: 4 hours n=9, 8 hours n=9, 12 hours n=8, 24 hours n=7; Daw Island, charged: 4 hours n=8, 8 hours n=9, 12 hours n=9, 24 hours n=9, 34 hours=3, noncharged: 4 hours n=9, 8 hours n=9, 12 hours n=9, 24 hours n=9, 34 hours=3). An * indicates significance between treatments (α=0.05) and for treatments at each time interval.

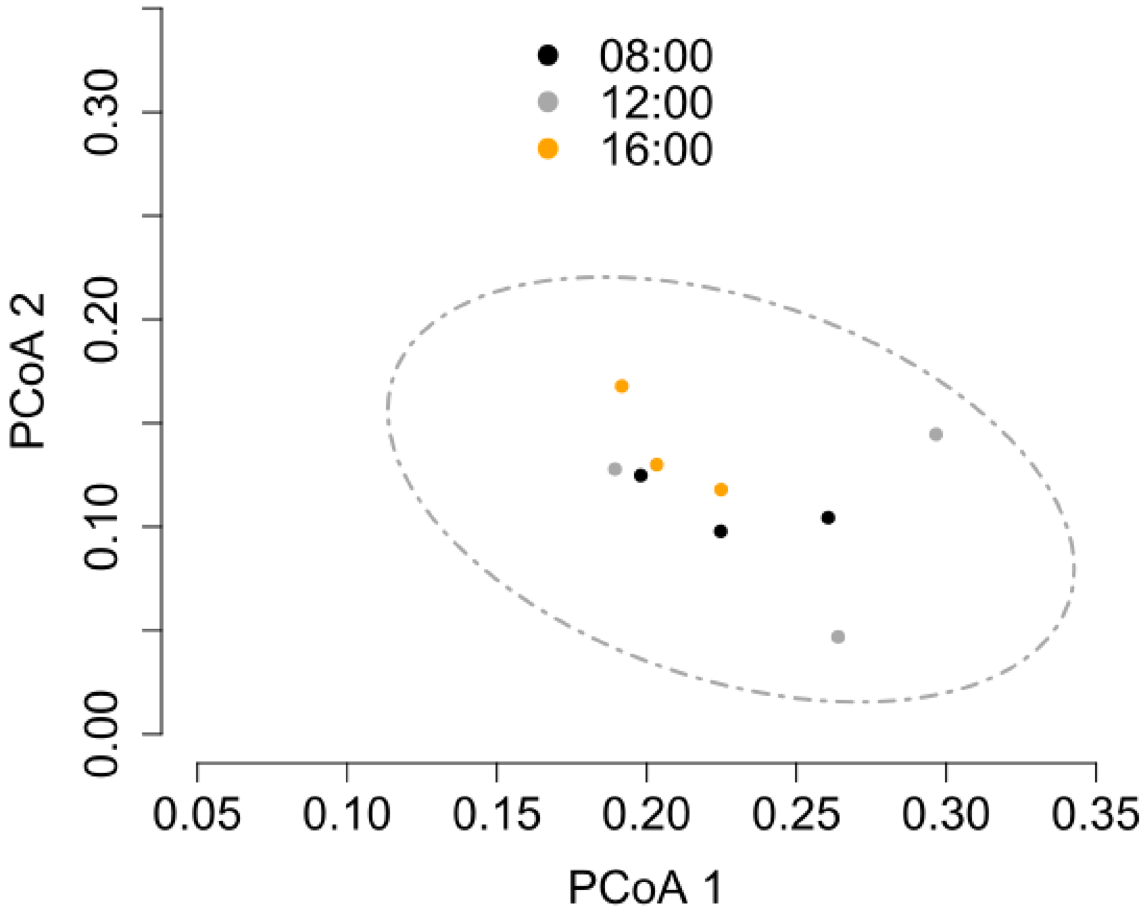

Figure S2. A detailed view of the Ashmore Reef principal coordinate analysis plot for active filtration by time of collection on the sample day.

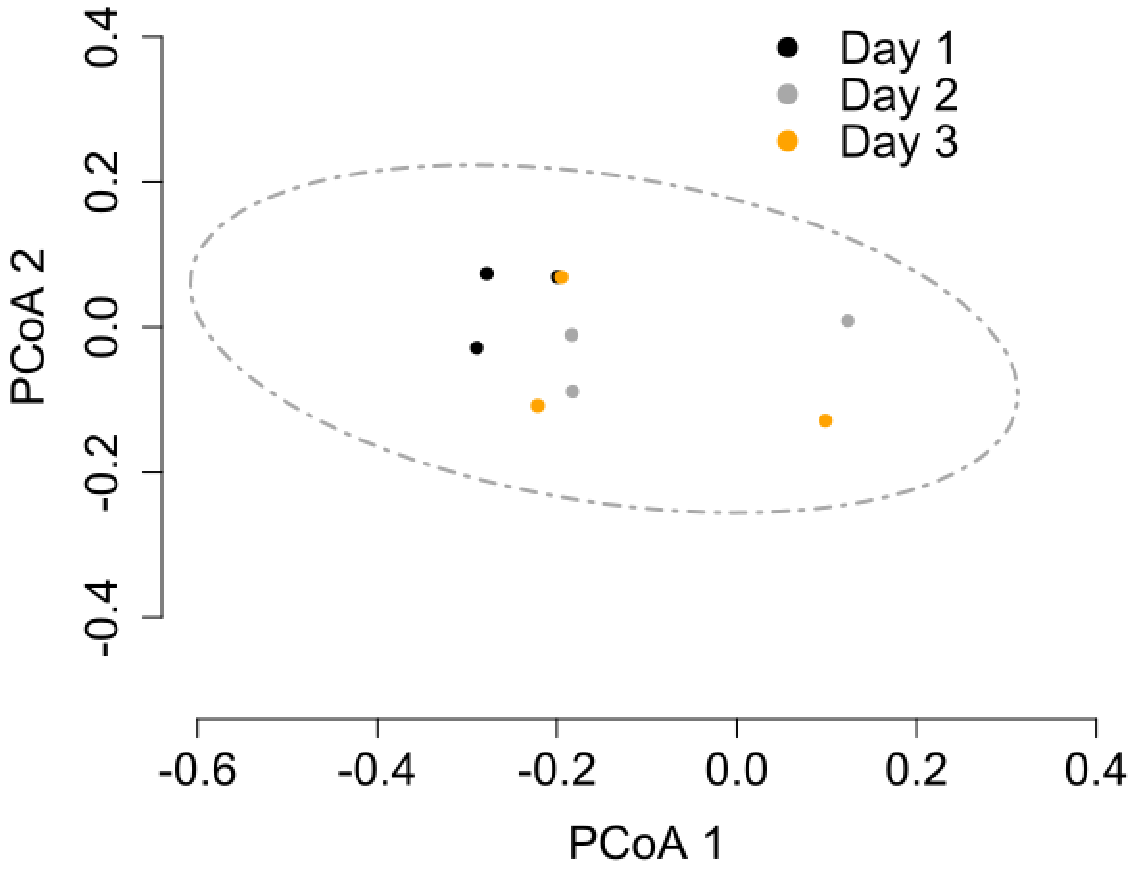

Figure S3. A detailed view of the Daw Island principal coordinate analysis plot for active filtration by day of sample collection.
