# Supplementary material for: Passive eDNA collection enhances aquatic biodiversity analysis
Source: Commun Biol. 2021 Feb 22;4:236. doi: 10.1038/s42003-021-01760-8 (PMC7900116; doi:10.1038/s42003-021-01760-8)
Supplement: Supplementary file 3 — Description of Supplementary Files [file 42003_2021_1760_MOESM3_ESM.pdf]

Description of Additional Supplementary Files

File Name: Supplementary Data 1

Description: Assigned taxonomy for detected Ashmore Reef fishes, including Codes for Australian Aquatic Biota (CAAB), site, location, date, identities, Genbank accession number, sequence, link to accessible data, number of reads and primer.

File name: Supplementary Data 2

Description: Assigned taxonomy for detected Daw Island fishes, including Codes for Australian Aquatic Biota (CAAB), site, location, date, identities, Genbank accession number, sequence, link to accessible data, number of reads and primer.
