# Supplementary material for: Passive eDNA collection enhances aquatic biodiversity analysis
Source: Commun Biol. 2021 Feb 22;4:236. doi: 10.1038/s42003-021-01760-8 (PMC7900116; doi:10.1038/s42003-021-01760-8)
Supplement: Supplementary file 6 — Reporting Summary [file 42003_2021_1760_MOESM6_ESM.pdf]

nature research

Corresponding author(s): Cindy Bessey

Last updated by author(s): Dec 21, 2020

Reporting Summary

Nature Research wishes to improve the reproducibility of the work that we publish. This form provides structure for consistency and transparency in reporting. For further information on Nature Research policies, see our Editorial Policies and the Editorial Policy Checklist.

Statistics

For all statistical analyses, confirm that the following items are present in the figure legend, table legend, main text, or Methods section.

n/a	Confirmed	
☐	☒	The exact sample size (*n*) for each experimental group/condition, given as a discrete number and unit of measurement
☐	☒	A statement on whether measurements were taken from distinct samples or whether the same sample was measured repeatedly
☐	☒	The statistical test(s) used AND whether they are one- or two-sided<br>*Only common tests should be described solely by name; describe more complex techniques in the Methods section.*
☐	☒	A description of all covariates tested
☐	☒	A description of any assumptions or corrections, such as tests of normality and adjustment for multiple comparisons
☐	☒	A full description of the statistical parameters including central tendency (e.g. means) or other basic estimates (e.g. regression coefficient) AND variation (e.g. standard deviation) or associated estimates of uncertainty (e.g. confidence intervals)
☐	☒	For null hypothesis testing, the test statistic (e.g. *F*, *t*, *r*) with confidence intervals, effect sizes, degrees of freedom and *P* value noted<br>*Give P values as exact values whenever suitable.*
☐	☒	For Bayesian analysis, information on the choice of priors and Markov chain Monte Carlo settings
☐	☒	For hierarchical and complex designs, identification of the appropriate level for tests and full reporting of outcomes
☐	☒	Estimates of effect sizes (e.g. Cohen's *d*, Pearson's *r*), indicating how they were calculated

Our web collection on statistics for biologists contains articles on many of the points above.

Software and code

Policy information about availability of computer code

Data collection	Illumina sequencing using a 300 cycle MiSeq® V2 Reagent Kit and standard flow cell
Data analysis	Python 2.7.13: OBITools 1.2.11 (https://pythonhosted.org/OBITools/), R (version 2.14.0; R Development Core Team 2011)

For manuscripts utilizing custom algorithms or software that are central to the research but not yet described in published literature, software must be made available to editors and reviewers. We strongly encourage code deposition in a community repository (e.g. GitHub). See the Nature Research guidelines for submitting code & software for further information.

Data

Policy information about availability of data

All manuscripts must include a data availability statement. This statement should provide the following information, where applicable:

- Accession codes, unique identifiers, or web links for publicly available datasets
- A list of figures that have associated raw data
- A description of any restrictions on data availability

A spatial representation of the eDNA survey results is available through the Atlas of Living Australia at https://collections.ala.org.au/public/show/dr16844.

Raw sequences, bioinformatic script, reference database, and the final datasets are available on the CSIRO Data Access Portal at https://data.csiro.au/collections/collection/CIcsiro:46025v1.

Field-specific reporting

Please select the one below that is the best fit for your research. If you are not sure, read the appropriate sections before making your selection.

☐ Life sciences ☐ Behavioural & social sciences ☒ Ecological, evolutionary & environmental sciences

For a reference copy of the document with all sections, see nature.com/documents/nr-reporting-summary-flat.pdf

Ecological, evolutionary & environmental sciences study design

All studies must disclose on these points even when the disclosure is negative.

Study description	Details of our nested study design are included in the manuscript. Briefly, to evaluate the viability of passively collecting eDNA, we submerged filter membranes approximately one meter below the surface in a pearl frame (n=150). To evaluate if submersion time affected collection of eDNA, we retrieved the filter membranes after different time intervals. We also investigated two different membrane materials. All treatments were done in triplicate over four days (June 17 to 20, 2019) at a tropical marine site (n=72 membranes) and five days (Jan 29 to Feb 2, 2019) at a temperate marine site (n=78 membranes). We then compared the species diversity obtained from passive eDNA collection to that achieved by the conventional method of actively filtering water samples. We collected nine 1 L surface water samples in sterile containers at each site and filtered the water over nine non-charged cellulose membranes using a peristaltic pump.
Research sample	We chose to analyse fish eDNA because this is one of the most common assessments made in aquatic eDNA metabarcoding studies with a substantial reference database for taxonomic assignment.
Sampling strategy	We compared the species diversity obtained from passive eDNA collection to that achieved by the conventional method of actively filtering nine 1L surface water samples at each site. We chose nine 1 L actively filtered water samples for comparison because this level of replication is rarely achieved by the majority of aquatic eDNA metabarcoding fish studies.
Data collection	Filter membranes were retrieved using gloved hands and sterile tweezers by Cindy Bessey at Daw Island, and by Cindy Bessey and Todd Stewart at Ashmore Reef.
Timing and spatial scale	Data collection at Ashmore Reef occurred over June 17 to 20, 2019. Triplicate membranes were retrieved after four, eight, 12, and 24 hours of deployment. Data collection at Daw Island occured over January 29 to February 2, 2019. Triplicate membranes were retrieved after four, eight, 12, 24 hours of deployment, and on the last day we also deployed triplicate membranes for 34 hours.
Data exclusions	All positive controls amplified multiple reads identifying dhufish with 100% identity. However, 36 reads of the positive control showed up in two Ashmore Reef samples. Therefore, to ensure a conservative approach to detection efficiency, we required a minimum of 40 reads to count a fish species as present.
Reproducibility	The experiment was repeated twice, once in tropical water and once in temperate waters. Both attempts were successful.
Randomization	Filter membranes were placed in the deployment frame to ensure all treatments were completely submerged and had equal exposure to the marine environment.
Blinding	All laboratory samples were labeled as a number and processed in the same fashion.

Did the study involve field work? ☒ Yes ☐ No

Field work, collection and transport

Field conditions	Sampling was conducted in the tropical waters of Ashmore Reef (June 17 to 20, 2019; average water temperature for June is ~25C) and the temperate waters surrounding Daw Island (Jan 29 to Feb 2, 2019; average water temperature for June is ~18C).
Location	At Ashmore Reef, sampling took place in surface waters at the vessel mooring site (122°58.99′ E, 12°14.27′ S), where the depth was less than 10 meters. Sampling at Daw Island took place in the surface waters at the vessel mooring site (124°07.86′ E, 33°51.01′ S), where the depth was less than 20 meters.
Access & import/export	All samples were taken from the boat at the mooring site.
Disturbance	Our experiments were non-invasive and caused no disturbance.

Reporting for specific materials, systems and methods

We require information from authors about some types of materials, experimental systems and methods used in many studies. Here, indicate whether each material, system or method listed is relevant to your study. If you are not sure if a list item applies to your research, read the appropriate section before selecting a response.

Materials & experimental systems

n/a	Involved in the study
☒	☐ Antibodies
☒	☐ Eukaryotic cell lines
☒	☐ Palaeontology and archaeology
☒	☐ Animals and other organisms
☒	☐ Human research participants
☒	☐ Clinical data
☒	☐ Dual use research of concern

Methods

n/a	Involved in the study
☒	☐ ChIP-seq
☒	☐ Flow cytometry
☒	☐ MRI-based neuroimaging
